# The Donkey Milk in Infant Nutrition

**DOI:** 10.3390/nu14030403

**Published:** 2022-01-18

**Authors:** Enrico Bertino, Massimo Agosti, Chiara Peila, Marinella Corridori, Roberta Pintus, Vassilios Fanos

**Affiliations:** 1 Neonatal Care Unit of the University, City of Health and Science Hospital, 10100 Turin, Italy; chiara.peila@unito.it; 2 Neonatal Intensive Care Unit, Human and Child Department, Ospedale “F. Del Ponte”, 21100 Varese, Italy; massimo.agosti@uninsubria.it; 3 Hygeia Press, Quartu S. Elena, 09045 Cagliari, Italy; m.corridori@hygeiapress.com; 4 Neonatal Intensive Care Unit, University Hospital of Cagliari, 09123 Cagliari, Italy; vafanos@tiscali.it (R.P.); gomberta@icloud.com (V.F.); 5 Department of Surgery, University of Cagliari, 09124 Cagliari, Italy

In the history of the world, the donkey has always had a special place next to humans since the time of its domestication, about 10,000 years ago. Its history is connected to that of humans, enough to be mentioned in the literature, in religious books, and mythological poems.

Donkey milk occupied a prominent role in ancient times, and today it comes back powerfully as a functional food for the human nutrition of the third millennium. In the ancient world, it was used for the nutrition of newborns through toy-shaped bottles. Today, it is a food that arouses increasing interest, and it is projected to be in the future of human nutrition.

Different narratives can be cited to demonstrate that the story of the donkey is inextricably linked to that of humans. Donkey milk in cosmetic use can count on four famous testimonials: Cleopatra, Poppea, Messalina, and Paolina Bonaparte. It is said that Cleopatra, queen of Ancient Egypt, used to immerse herself in donkey milk to keep her beauty intact and preserve the splendor of her skin. Legend says that 700 donkeys were needed to provide her with the amount of milk necessary for her daily beauty baths. It seems that it had been done so even by Poppea, the second wife of the Roman emperor Nero, as referred by Pliny the Elder, describing the virtues of this milk for the skin: “Donkey milk is believed to eliminate wrinkles from the skin of the face and make it softer and whiter, and some women are known to treat their cheeks seven times a day, paying close attention to this number. It was Poppea, wife of the Roman emperor Nero, who started this fashion, making use of it even for the baths, and for this reason, she brought along herds of donkeys when she traveled”. Messalina, Roman empress, the wife of Emperor Claudius I (10 BC–54 AD), used facial masks with donkey-milk-soaked bread slices. Pauline (1780–1825), sister of Napoleon Bonaparte, would have used donkey milk to maintain the beauty of her skin.

In addition to its use in cosmetics, donkey milk has always been used since ancient times. In fact, already Herodotus in the 5th century B.C. mentions it as a nutritious drink. Hippocrates (460–370 BC.), the father of medicine, was the first to describe the medicinal virtues of donkey milk. He prescribed donkey milk for numerous ailments, such as liver problems, edemas, nosebleeds, poisonings, infectious diseases, the healing of sores, and fevers. In Roman times, donkey milk was used as a universal remedy: Pliny the Elder (23–79 AD), in his encyclopedic work Naturalis Historia, has widely described its health benefits. In particular, Pliny writes about 54 medicinal uses of donkey milk, ranging (spacing) from its use as an anti-venom or as a relief for external irritations (itching) to the use of it in a pomade (ointment) for the eyes. He states that donkey milk is the most effective as a medicine, followed by cow’s milk, and then goat’s milk.

During the Renaissance, donkey milk was the subject of a first real scientific consideration by the wise men of the time, when Francis I, king of France, on the advice of his doctors, used donkey milk to recover from a long illness. There are various testimonials concerning the effectiveness of donkey milk. The famous French naturalist Georges-Louis Leclerc (1707–1788) underlined the benefits of donkey milk in his *Histoire Naturelle*.

In the XIX century, still in France, the practice of latching orphaned babies directly to the donkey nipple to be breastfed was spread through the work “Hôpital des Enfants Assistés” of Dr. Parrot. In the nineteenth century, in large European cities, it was easy to come across merchants who directly sold donkey milk. The elegant society of the time consumed this precious drink regularly, while low-income families reserved it only for sick children or tired and weak older adults. During this period, donkey milk begins to be used regularly for breastfeeding. Until the XX century, donkey milk was sold to feed orphaned babies, debilitated children, sick people, and elders. For this reason, several asinerias arose in Italy, Belgium, Germany, and Switzerland.

Donkey milk was used until the beginning of the XX century as a substitute for breast milk. The testimony of Prof. Charles Porcher (1827–1933) of the Veterinary School of Lyon, France, in 1928 shows that the practice was still prevalent, but to a lesser extent, after the First World War: “It seems that donkey milk has returned as food for early infancy in case, in particular, in which the child is of rather delicate health. Not that the donkey milk has been completely abandoned but, while about 25–30 years ago, donkeys reared for their milk with which the infants were fed, were easily found in cities, one can state that, by then this practice had, so to speak, completely disappeared”.

In the last decades, the scientific community has rediscovered the importance of donkey milk in human nutrition. Its composition is more similar to human milk compared to cow milk (lactose, lipid, and protein profile): Thanks to its chemical and nutritional profile, it is an excellent food used in the medical and food field. Thanks to its clinical tolerance, palatability and nutritional adequacy associated to the low levels of caseins and other proteins with high immunogenic power, donkey milk is particularly suitable for children suffering from cow’s milk protein allergy (CMPA).

Another important aspect of donkey milk in the medical field is its ability to regulate intestinal microflora. Among the functional proteins found in donkey milk, we want to mention lysozyme and lactoferrin, known for their antimicrobial activity. Lysozyme activity is intense, especially against Gram-positive bacteria. It also promotes the growth of the intestinal flora, constitutes a stimulating factor, and has anti-inflammatory functions. The lactoferrin content in donkey milk is intermediate between the value reported in cow’s milk and the highest value reported in human milk. Lactoferrin inhibits the growth of iron-dependent bacteria present in the gastrointestinal tract. Lactoferrin also protects against viral diseases, including those caused by coronavirus.

In donkey milk, as in mare’s milk, there are significant quantities of other bioactive molecules, such as sialylated oligosaccharides, in higher quantities than those found in bovine milk. Their presence in human milk has an anti-infective action and constitutes a factor that stimulates the immune system of the newborn. Another action is to prevent cardiovascular disease: Thanks to the presence of omega-3 fatty acids, a regular intake of donkey milk plays a preventive action towards the cardiovascular districts, preventing the formation of atherosclerotic plaques.

Experimental data on animal models observed that donkey milk affects glucose metabolism in a manner more similar to human milk than cow milk and that might have beneficial effects by changing energy homeostasis in favour of fatty acid oxidation, thereby reducing fat storage. Yvon et al. also observed that donkey milk consumption exerts anti-inflammatory properties by normalizing antimicrobial peptides levels in Paneth’s cells, so the authors speculate about its possible use as dietetic intervention in patients with Crohn’s disease. Moreover, recent data suggest that donkey colostrum and mature milk inhibit the growth and metastasis of mouse 4T1 tumors by inducing apoptosis. Thus, anticancer properties could be hypothesized for the future as well.

Considering the mentioned benefits of donkey milk, in particular, its use in the treatment of subjects with severe allergy to cow’s milk proteins and its possible use in prevention/treatment of cardiovascular, autoimmune, and inflammatory diseases, it has also been speculated that it could be suitable for the production of human milk fortifiers for feeding preterm infants.

In previous studies, it has been observed that the use of a novel donkey-milk-based fortifier seems to improve feeding tolerance in preterm infants when compared to a bovine-derived fortifier, with similar short-term auxological outcomes.

Data reported in this Special Issue deepen the knowledge of the short- and long-term effects of the use of donkey milk as a fortifier of human milk for feeding premature babies. The paper of Giribaldi et al. describes the differences in urinary metabolomic profile of preterm infants receiving Human Milk with either bovine- or donkey-milk-based fortifiers as a consequence of different quality of the nutrients provided by the fortifiers [1]. The study of Cresi et al. provides additional data on better feeding tolerance in preterm infants fed with a donkey-milk-based human milk fortifier. In this study, the use of donkey fortifiers reduced the frequency of gastroesophageal reflux in infants showing clinical signs of gastroesophageal reflux and cardiorespiratory symptoms associated to feeding intolerance [2]. Finally, the two studies of Peila et al. demonstrated no difference for the donkey-milk-derived fortifier compared to standard bovine-derived fortifier regarding long-term auxological and neurodevelopmental outcomes [3,4].

Increasing scientific evidence and interest in donkey milk allow us to highlight, according to a recent The Telegraph article, that donkey milk could be “the next big thing” in infant nutrition.
